# Potential molecular mechanism in self-renewal is associated with miRNA dysregulation in sacral chordoma – A next-generation RNA sequencing study

**DOI:** 10.1016/j.heliyon.2022.e10227

**Published:** 2022-08-13

**Authors:** Arpad Bozsodi, Beata Scholtz, Gergo Papp, Zoltan Sapi, Adam Biczo, Peter Pal Varga, Aron Lazary

**Affiliations:** a National Center for Spinal Disorders, Buda Health Center, Királyhágó u. 1-3, Budapest, H-1126, Hungary; b School of PhD Studies, Semmelweis University, Üllői út 26, Budapest, H-1085, Hungary; c Genomic Medicine and Bioinformatic Core Facility, Dept. of Biochemistry and Molecular Biology, Faculty of Medicine, University of Debrecen, Nagyerdei krt. 98, Debrecen, H-4032, Hungary; d 1st Department of Pathology and Experimental Cancer Research, Semmelweis University, Üllői út 26, Budapest, H-1085, Hungary; e Department of Spine Surgery, Department of Orthopaedics, Semmelweis University, Királyhágó u. 1-3, Budapest, H-1126, Hungary

**Keywords:** Chordoma, Next-generation sequencing, miRNA, mRNA, Transcriptome profile

## Abstract

**Background:**

Chordoma, the most frequent malignant primary spinal neoplasm, characterized by a high rate of recurrence, is an orphan disease where the clarification of the molecular oncogenesis would be crucial to developing new, effective therapies. Dysregulated expression of non-coding RNAs, especially microRNAs (miRNA) has a significant role in cancer development.

**Methods:**

Next-generation RNA sequencing (NGS) was used for the combinatorial analysis of mRNA-miRNA gene expression profiles in sacral chordoma and nucleus pulposus samples. Advanced bioinformatics workflow was applied to the data to predict miRNA-mRNA regulatory networks with altered activity in chordoma.

**Results:**

A large set of significantly dysregulated miRNAs in chordoma and their differentially expressed target genes have been identified. Several molecular pathways related to tumorigenesis and the modulation of the immune system are predicted to be dysregulated due to aberrant miRNA expression in chordoma. We identified a gene set including key regulators of the Hippo pathway, which is targeted by differently expressed miRNAs, and validated their altered expression by RT-qPCR. These newly identified miRNA/RNA interactions are predicted to have a role in the self-renewal process of chordoma stem cells, which might sustain the high rate of recurrence for this tumor.

**Conclusions:**

Our results can significantly contribute to the designation of possible targets for the development of anti-chordoma therapies.

## 1 Introduction

Chordoma is the most frequent primary malignant spinal tumor [1], often developing at the skull base and the sacrum where the surgical resection is challenging. It is a typical low-grade malignant lesion characterized by slow, locally aggressive growth with variable distant metastatic capacity, but a very high rate of local recurrence [2]. En bloc resection with wide margins gives the best oncological outcome [3, 4] but this is often extremely difficult or impossible to achieve due to the large tumor size, the surrounding vital structures, and the high probability of severe neurological impairment. To improve the oncological and the clinical outcome, reduce the high rate of local recurrences after tumor resection, and extend the survival, new, effective adjuvant therapeutic options would be needed because chordomas are highly resistant to the existing chemotherapy and radiotherapy options [2]. A major obstacle to developing novel anti-chordoma agents is our limited knowledge about the molecular mechanism of chordoma tumorigenesis.

Chordoma generally manifests between the age of 40–60, and it is considered to originate from the malignant transformation of notochordal cell remnants in the axial skeleton [5]. During development, the embryonic notochord disappears almost completely, and the only notochord-derived differentiated cells in humans are the nucleus pulposus (NP) cells of the intervertebral discs. Neither the differentiation process of human NP cells nor the biogenesis of chordoma tumor cells has been characterized fully [5, 6]. In recent years, the transcription factor T (brachyury) and its genetic polymorphism were described as critical, chordoma-specific oncogenic drivers [7, 8]. A cancer vaccine targeting the brachyury was recently developed [9], but its clinical efficacy in chordoma patients is not yet known. Activation of the PI3K/AKT/TSC1/TSC2/mTOR signaling pathway [10], overexpression of receptor tyrosine kinases (such as EGFR), as well as activation of IGF1R were described in chordoma and were considered as targets for tailored therapy [11, 12]. Despite these discoveries, sporadic case reports or small case series have shown that even targeted chemotherapy against these oncogenic pathways provided partial tumor response at best [13, 14, 15, 16].

The failure of the classic and novel targeted chemotherapeutic agents against chordoma might be related to its complex and heterogeneous oncogenesis, which is characterized by a modest somatic mutation rate, but a high genome instability, impacting just certain chromosomal arms or resulting in chromothripsis [17, 18]. Importantly, these changes may impact not only protein-coding genes but noncoding RNA genes as well, which regulate multiple molecular networks at the transcriptional or post-transcriptional level. MicroRNAs (miRNA) and long non-coding RNAs (lncRNA) are critical regulators of normal cell and tissue homeostasis [19], and as miRNAs have been shown to have important roles in tumorigenesis, they are extensively studied as new targets of anticancer drug development [20, 21, 22].

In recent years, a growing body of evidence has supported the possible role of altered miRNA expression in the development and progression of chordoma [23, 24, 25, 26, 27, 28, 29, 30, 31, 32, 33, 34, 35], however, the published studies are characterized by a wide variety of the used biological samples and research methods. The latest development in total transcriptome profiling techniques, such as next-generation sequencing (NGS), and the related bioinformatics tools have provided the possibility to carry out the comparative, quantitative determination of the detailed miRNA expression patterns in different tumors in parallel to their gene expression profiling [36, 37]. Using the technique, complex RNA-based regulatory networks and molecular pathways can be identified revealing possible new molecular targets.

In the present study, next-generation small RNA and mRNA sequencing was used in parallel to determine the altered miRNA expression profile of sacral chordoma, compared to nucleus pulposus cells, and to predict their effect on the miRNA-mRNA networks in chordoma.

## 2 Materials and methods

### 2.1 Patients and tissue samples

8 chordoma (CH) and 8 nucleus pulposus (NP) tissue samples were collected from surgically treated Caucasian patients. Tumor samples were obtained from 3 male and 5 female subjects (mean age: 61.1 ± 12.5y). All chordomas were localized to the sacrum and histologically classified as classic chordomas. Five cases were recurrent tumors, and none of the patients had received any adjuvant treatment before the surgery. The clinical characteristics of the patients are summarized in Supplementary Table 1. The chordoma samples were frozen in liquid nitrogen immediately after removal and stored at −80 °C until processing. NP tissues were surgically removed from 8 patients (mean age: 60.3 ± 10.5y) diagnosed with degenerative disc disease (Pfirrmann grade III), undergoing lumbar intervertebral fusion surgery. All patients were informed and signed consent of approval for using their tissue samples for the study. The study was approved by the Scientific and Research Ethics Committee of the Medical Research Council (49777/2012/EKU (751/PI/12)).

### 2.2 NP tissue cultures

Previously, we tested RNA isolation directly from surgically obtained NP tissues, but the quality and quantity of RNA were inadequate for next-generation sequencing, despite extensive optimization of the isolation protocol. Therefore, we established primary NP cell cultures from the NP tissue samples, applying the explantation method without any enzymatic digestions. NP cell cultures were maintained in Dulbecco's Modified Eagle's Medium/Ham's Nutrient Mixture F12 (Sigma-Aldrich) containing 20% fetal bovine serum (Sigma-Aldrich), and penicillin-streptomycin (100 U and 100 mg/mL, respectively). Cells were cultured in 25 cm^2^ tissue culture flasks at 37 °C under a humidified atmosphere containing 5% (v/v) CO_2_. The NP cells had a small polygonal shape with short processes in the monolayer cultures, which is the usual appearance of these cells in vitro [38].

### 2.3 RNA isolation, quantification, and quality control

Snap-frozen chordoma tissue samples were pulverized while frozen, the tissue powder was lysed immediately with TRIzolate Reagent (Sigma-Aldrich) (50 mg sample per 500 uL reagent), and the samples were homogenized with a rotor-stator homogenizer. An equal volume of chloroform was added to the homogenates and vortexed, and the aqueous phase was separated using Phase Lock Gel™ (QuantaBio) (PLG 15 ml Heavy) tubes. RNA was isolated from the aqueous phase with the Direct-Zol RNA Miniprep kit (Zymo Research), according to the manufacturer's instructions, without DNase I treatment. RNA was prepared from the NP cell cultures with TRIzolate Reagent and Direct-Zol RNA Miniprep kit, according to the manufacturer's instructions. RNA quality was checked with Nanodrop 1000 spectrophotometer and Agilent 2100 Bioanalyzer – the data is summarized in Supplementary Table 2.

### 2.4 Library preparation and sequencing

For RNA-seq, sequencing libraries were prepared with the Illumina TruSeq RNA SamplePrep v2 kit and for small RNA-seq, sequencing libraries were prepared with the Illumina TruSeq Small RNA SamplePrep kit, according to the manufacturer's instructions. All libraries were indexed, allowing multiplexed sequencing. The RNA-seq libraries were sequenced as 2 × 100 bp paired-end reads, and the small RNA-seq libraries were sequenced as 1 × 50 bp single-end reads on Illumina HiSeq 2000. Raw data were preprocessed with the CASAVA 1.8.2 software (converts∗.bcl files into∗.fastq.gz files = compressed FASTQ files) and resulted in the demultiplexed, fastq.gz files used in all subsequent analyses.

### 2.5 Bioinformatic analysis of RNA next-generation sequencing data

For most bioinformatic analyses either the public Galaxy Main server or the Galaxy Mississippi server was used (https://usegalaxy.org/, https://mississippi.snv.jussieu.fr/). The sequencing quality of the raw data was first checked with FASTQC. Data were preprocessed using Trimmomatic (RNA-seq) and Adapter clipping + Trimmomatic with a size selection of 19–25 nt (small RNA-seq). RNA-seq for one sample (CH4) was unsuccessful, likely due to an index sequence mixup or mislabeling during library preparation, resulting in a missing file after demultiplexing, but the small RNA-seq was successful for this sample as well. Based on the FASTQC-based quality control analysis, all small RNA-seq and RNA-seq reads were of high quality, and the libraries had the expected total read counts. Sequence length distribution plots of the small RNA-seq data revealed that the control (NP) sequencing libraries contained a higher ratio of longer RNA species (presumably snoRNA or tRNA fragments) and a population of shorter (14–18 bp) fragments. To ensure better normalization between chordoma and control sequencing libraries in the differential expression analysis, miRNA-seq data preprocessing included the selection of the 19–25 nt fragment population from all samples.

Preprocessed RNA-seq data were aligned to the hg19 reference genome using TopHat2, and the read count table was prepared with Featurecounts. Preprocessed small RNA-seq data were aligned to human miRNA hairpin sequences using Chimira [39], and the resulting plain counts table was used in the subsequent steps. Only miRNAs with a minimum average read count ≥5 in either the CH or the NP samples were considered for differential gene expression analysis. A value of 1 was added to every value in the Featurecounts tables to eliminate zero values. Differential gene expression analysis was done with EdgeR [40] using the read count tables, hierarchical clustering using Euclidean distance measure and average linkage were performed with ClustVis [41], and a heatmap of relative expression levels was generated with GraphPad Prism. Principal component analysis was performed with DESeq2. Since clustering analysis of RNA-seq data suggested that the NP11 nucleus pulposus sample is an outlier, the sample was left out of the differential gene expression analysis (data not shown). Details and parameter settings of the analysis steps and basic statistics of preprocessing and alignment are provided upon request.

### 2.6 miRNA target prediction and pathway analysis

Differentially expressed miRNAs were selected from the EdgeR results table, using the following filters: FDR (adjusted p-value) ≤ 0.05, |log2FoldChange| ≥ 3. The top upregulated or downregulated miRNAs were those with |log2FoldChange| ≥ 5. Differentially expressed mRNAs were selected from the EdgeR results table, using the following filters: FDR (adjusted p-value) ≤ 0.01, |log2FoldChange| ≥ 2, and dispersion ≤ 1.54. Genes without functional annotation and partial cDNA genes were removed from the list. Targets of the differentially expressed miRNAs were predicted with the Targetscan 7.1 algorithm (http://www.targetscan.org/vert_71/) [42]. Gene ontology (GO) annotations for functional classification of target genes were based on the Panther Classification System (http://pantherdb.org/) [43] and literature searches. Pathway analysis was done with the Consensus PathDB human, release 32 (gene set overrepresentation analysis) (http://cpdb.molgen.mpg.de/CPDB) [44].

### 2.7 Real-time quantitative PCR analysis and data processing

The expression of selected miRNAs and mRNAs was validated by real-time quantitative PCR (qPCR) method. TaqMan™ assay IDs and details of the RT-qPCR protocol are provided in the Supplementary Data. For miRNA data normalization we tested RNU44, RNU6B, miR-106b, and miR-30d, and selected RNU44 as the best reference gene based on the analysis with the NormFinder software [45]. For mRNA data normalization we tested GUSB, GAPDH, Cyclophilin A (PPIA), RPLP0, and HPRT1 as reference genes, and selected PPIA as the best reference gene. Samples were measured on StepOne™ Real-Time PCR System (Applied Biosystem®). Raw data analysis was performed using the StepOne Software v2.2.2 (Applied Biosystem®). Cutoff Ct values (limit of quantification) for each TaqMan® assay set at Ct = 37. Ct-s above this value, or “undetected” values were uniformly set to Ct = 40. Normalized expression values were calculated using the 2^−dCt^ method [46]. Statistical analysis was done with GraphPad Prism, using Mann-Whitney U-tests on the normalized expression values.

## 3 Results

### 3.1 Expression profile of miRNAs

**Figure 1:**
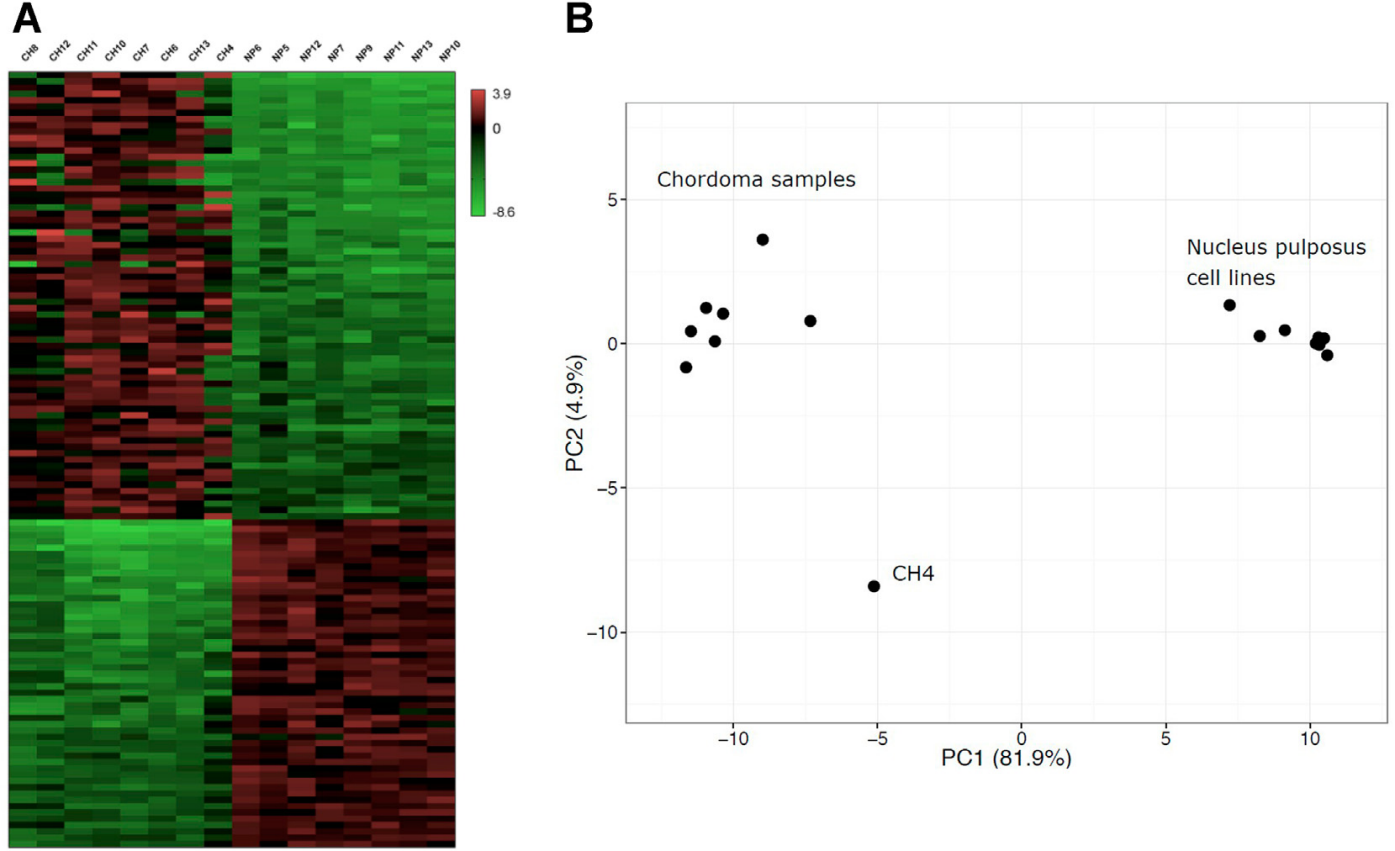
Differentially expressed miRNAs in chordoma tumors (CH) and nucleus pulposus (NP) cell lines. a) Heatmap of the 126 differentially expressed (DE) miRNAs in chordoma. Normalized expression values from Chimira/EdgeR analysis were scaled and log2 transformed. The color scale of the heatmap illustrates the relative expression levels of the DE miRNAs. Numerical representation and DE miRNA names are in Supplementary Table 4. b) Principal component analysis of the samples based on Chimira/DESeq2 analysis.

Our analysis identified 126 differentially expressed miRNAs, with 74 miRNAs being upregulated and 52 downregulated in chordoma (Figure 1a and Supplementary Table 3). The PCA analysis based on the miRNA differential expression profile demonstrated a clear separation of CH samples from NP samples and also identified the CH4 chordoma sample as an outlier (Figure 1b). It should be noted that clinical follow-up showed that the CH4 patient is still in complete remission, as opposed to the other chordoma patients who all succumbed to the disease or its complications. 52% (N = 66) of the dysregulated miRNAs were intragenic, and 39% of these (26 miRNAs) were co-regulated with their host genes. Interestingly, several downregulated ncRNAs clustered to chromosome 14 at the border of chromosomal bands 14q32.2 and 14q32.3, including miRNAs located between hsa-miR-493 and hsa-miR-409, the small nucleolar RNAs SNORD112-114, and the long intergenic noncoding RNAs MEG3/8/9.

### 3.2 miRNA target prediction and pathway analysis

**Figure 2:**
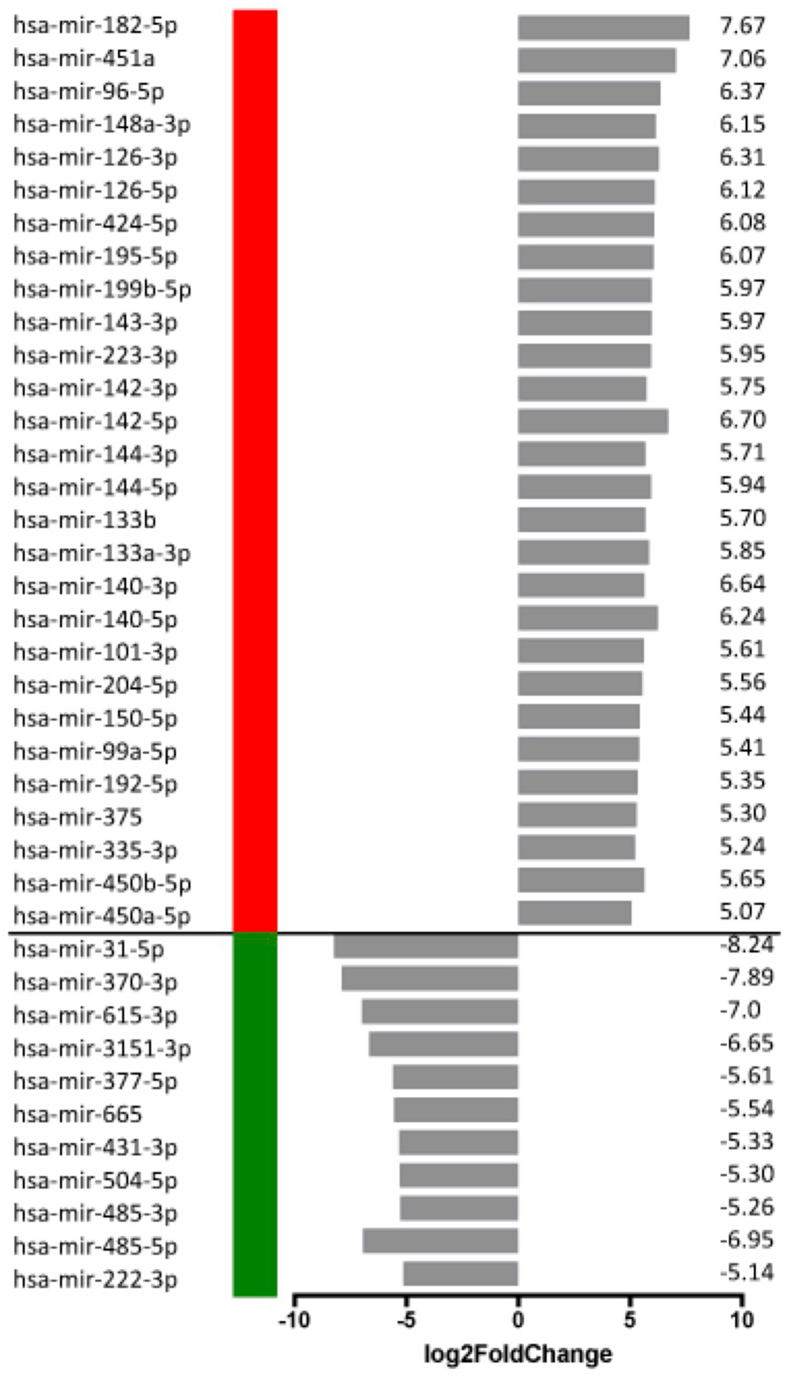
Top dysregulated miRNAs in chordoma. Relative expression (log2FoldChange) and direction of the dysregulation are represented (red: upregulated, green: downregulated miRNAs).

**Figure 3:**
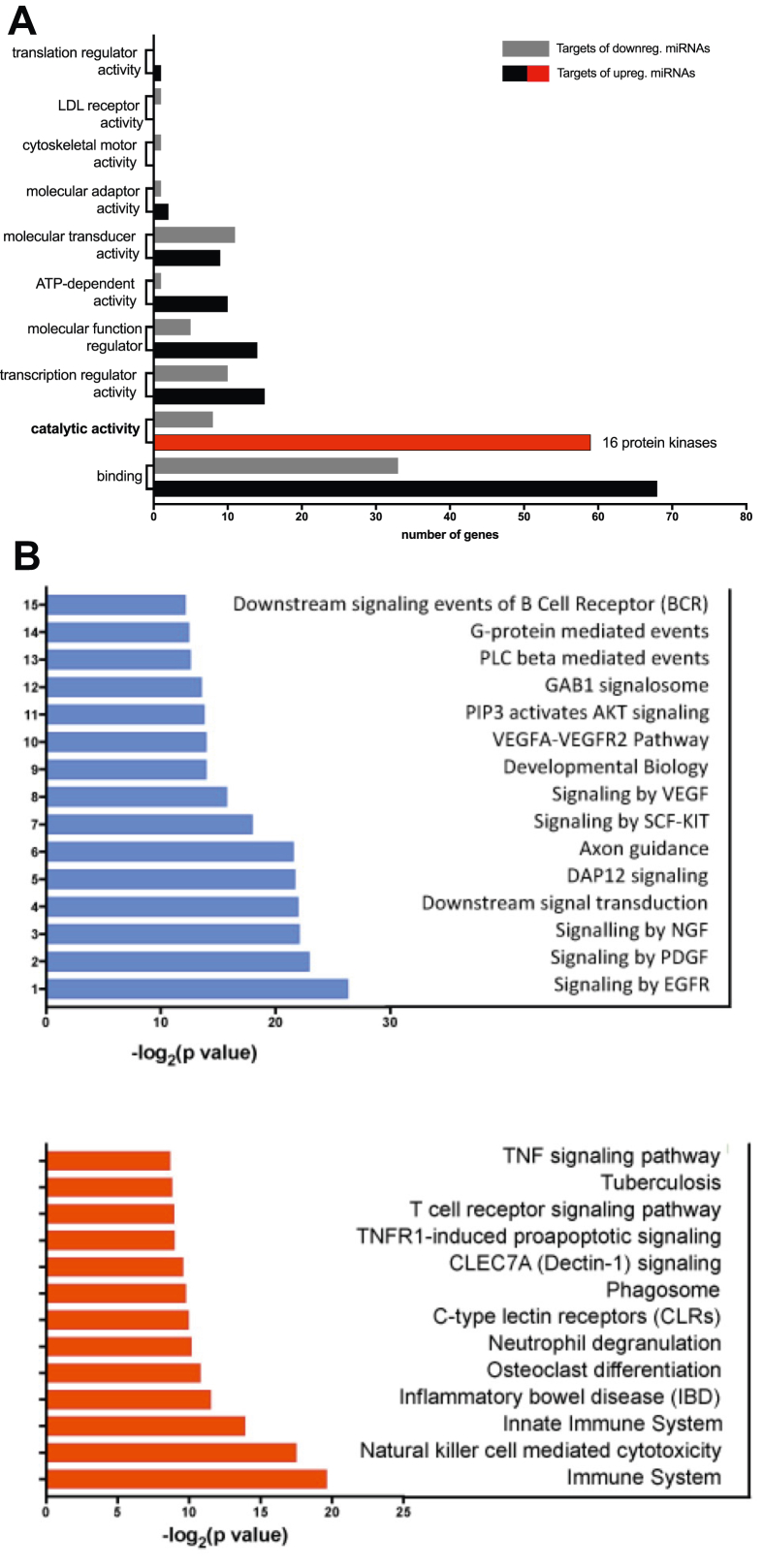
Results of functional classification analysis (a) and pathway analysis (b) of the predicted targets of upregulated and downregulated miRNAs in chordoma. (a) The protein function GO categories identified by Panther DB functional analysis (GO-Slim Molecular function) are shown. (b) Overrepresented pathways were identified with the ConsensusPath DB algorithm. The x-axis shows the -log2(p-value) of the enrichment. Top panel: Enriched pathways for downregulated genes predicted to be targeted by upregulated miRNAs. Bottom panel: Enriched pathways for upregulated genes predicted to be targeted by downregulated miRNAs.

Next, we selected the top upregulated and downregulated miRNAs for mRNA target prediction (Figure 2). Predicted target genes (based on Targetscan analysis) were filtered by their Cumulative weighted context++ scores (score ≤ −0.3) and overlapped with the list of significantly up- or downregulated genes from the RNA-seq analysis. Based on the literature, the majority of downregulated predicted target genes of upregulated miRNAs have anti-tumor activities – on the other hand, the upregulated genes targeted by downregulated miRNAs are mostly oncogenic (Supplementary Figure 1). Functional classification of the target genes based on Panther DB analysis revealed that both up- and downregulated miRNAs target transcription factors and components of signal transduction pathways. In addition, upregulated miRNAs may target regulators of chromatin structure and 16 protein kinases as well, as opposed to downregulated miRNAs, which do not target genes with chromatin-associated functions, and may target just a single protein kinase, ITK (Figure 3a, Supplementary Figure 1 and Supplementary Table 4). Overrepresentation analysis of predicted target genes identified different enriched pathways that may be regulated by the up – or downregulated miRNA sets (Figure 3b). Similarly to target gene functional classification, upregulated miRNAs are likely to target various signal transduction pathways, whereas downregulated miRNAs appear to target pathways related to immune response regulation. A notable target is the TNF signaling pathway, which was shown to activate metastatic and tumor-promoting inflammatory pathways in chordoma. We observed significant overexpression of TNF family members and several TNF receptors (TNFRSF1B/4/8/18) in the chordoma samples (data not shown). Interestingly, NFATC2 (nuclear factor of activated T-cells), a transcriptional regulator of TNF and TNF receptor expression in melanoma cell lines [47] is also overexpressed in the chordoma samples and may be targeted by the downregulated miRNA miR-665.

### 3.3 Identification of a possible self-renewal molecular mechanisms in chordoma

**Figure 4:**
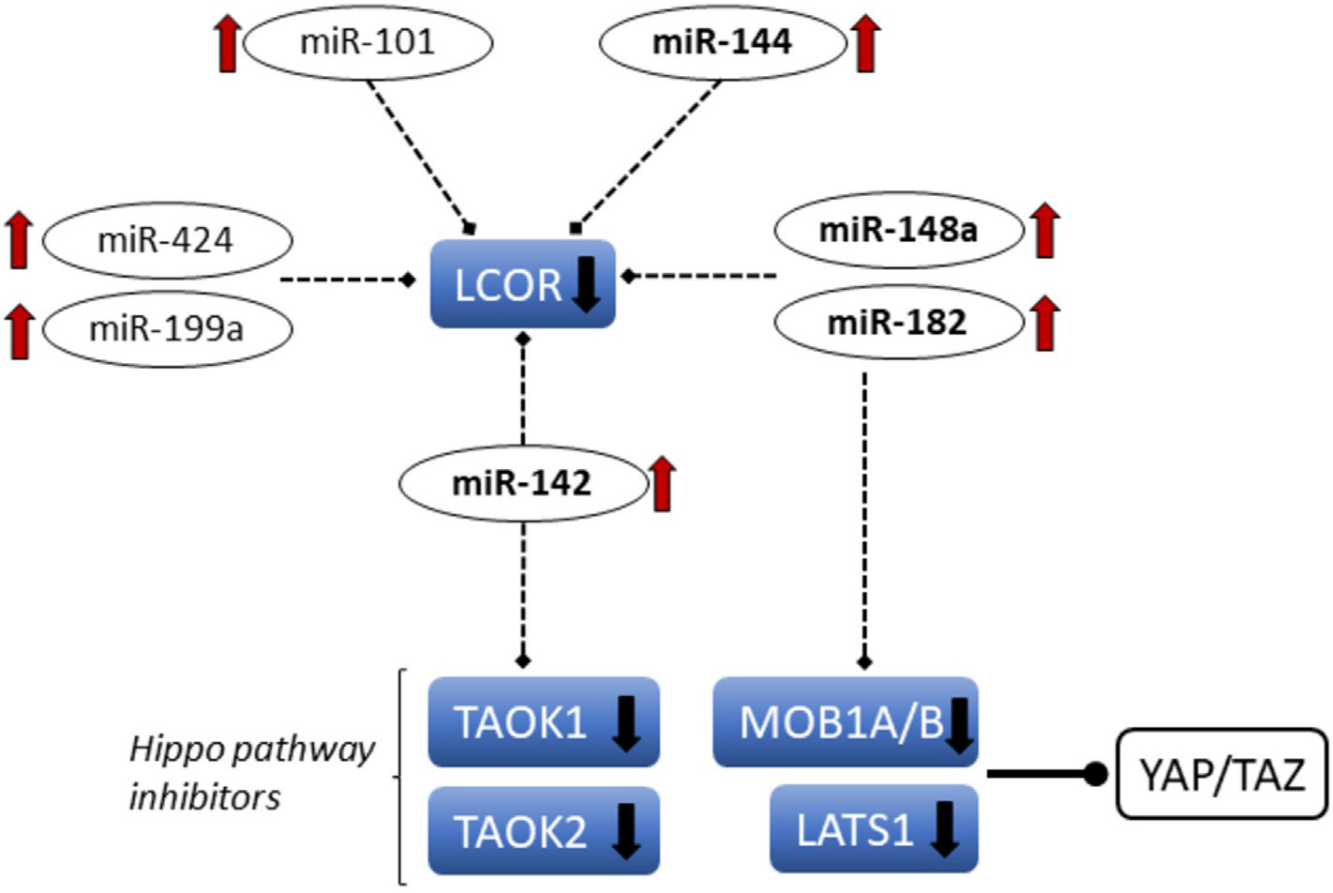
Predicted miRNA-mRNA regulatory network of self-renewal in chordoma. Bold arrows indicate the direction of RNA expression dysregulation: red = upregulated, black = downregulated. Upregulated miRNAs validated by RT-qPCR are in bold.

To identify the genes that are the most likely candidates for regulation by differentially expressed miRNAs in chordoma, we selected several dysregulated genes that may be targeted by multiple dysregulated miRNAs, and target genes with the best cumulative weighted context++ scores (score ≤ −0.6) (Supplementary Table 5). This approach suggested several previously validated regulatory mRNA/miRNA interactions between LCOR/miR-199a-5p, MITF/miR-182-5p/miR-101/miR-148a-3p, HIF3a/miR-485-5p, HMGA1/miR-142-3p, RASA1/miR-182-5p/miR-223-3p, RGS17/miR-182-5p and RICTOR/miR-142-3p. Functional annotation of the mRNA targets predicted a miRNA-mRNA network regulating the self-renewal potential of chordoma tumors, through the regulation of the transcription factor LCOR, and the potential activation of the Hippo pathway through targeting its regulators, TAOK and MOB1 (Figure 4).

### 3.4 qPCR validation of the dysregulated self-renewal pathway

**Figure 5:**
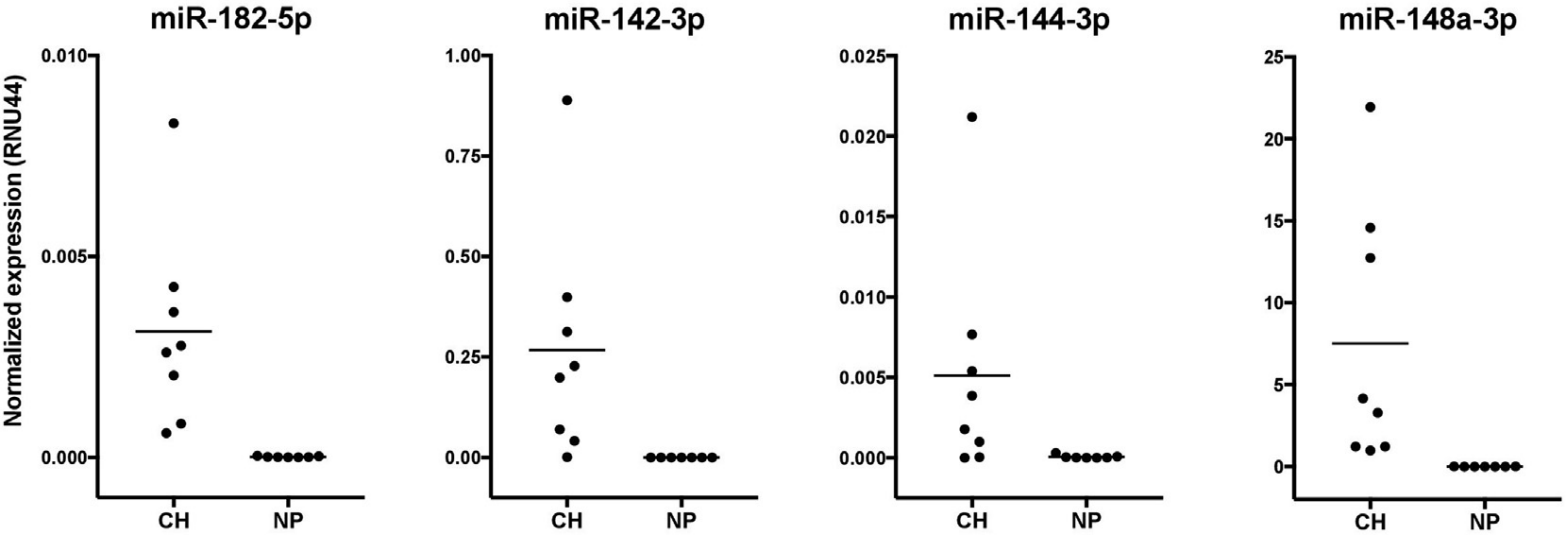
RT-qPCR validation of miRNA expression in chordoma (CH) and nucleus pulposus (NP) samples. Normalized expression was calculated with the 2^−dCt^ method, with RNU44 as the reference gene. All differences were statistically significant (Mann-Whitney test, p ≤ 0.001).

**Figure 6:**
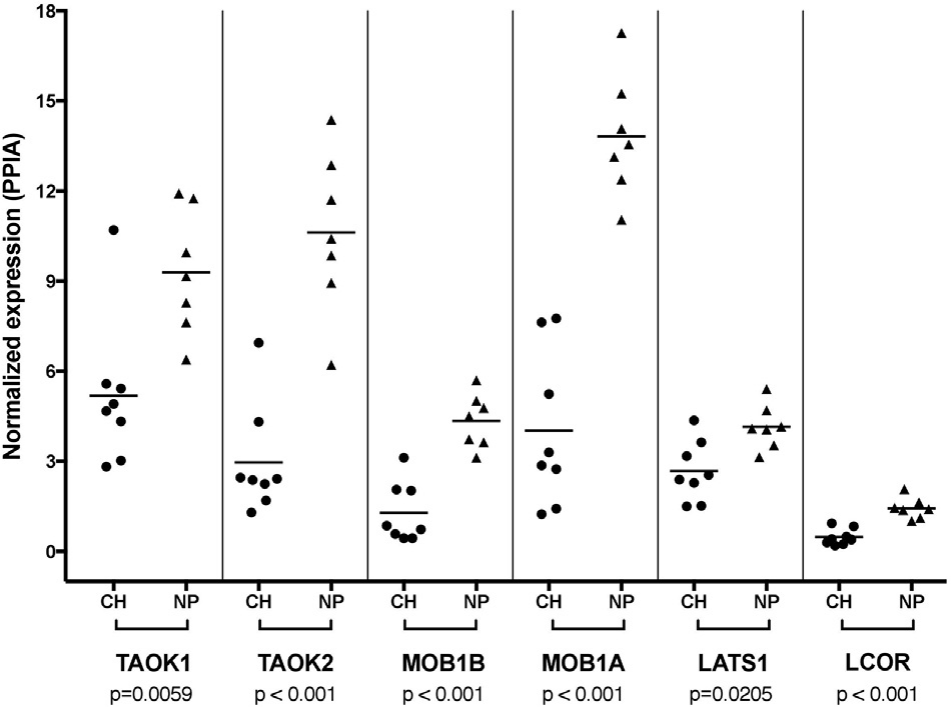
RT-qPCR validation of mRNA expression in chordoma (CH) and nucleus pulposus (NP) samples. Normalized expression was calculated with the 2^−dCt^ method, with PPIA as the reference gene (p-values are from Mann-Whitney tests).

We selected the 4 miRNAs and 6 mRNAs from the predicted self-renewal regulatory network for RT-qPCR validation. The qPCR analyses confirmed the significantly different expression and the reciprocal regulation of the selected miRNAs (Figure 5) and their target mRNAs (Figure 6) in chordoma vs. nucleus pulposus samples, similar to the results of the NGS analysis. We also validated the reciprocal regulation of NFATC2 and miR-665 (data not shown).

## 4 Discussion

In the present study, we used next-generation sequencing for the combined analysis of global miRNA and mRNA gene expression changes in sacral chordoma. This multi-omics approach and the subsequent detailed bioinformatic analysis predicted a novel molecular mechanism for the regulation of self-renewal in chordoma, never identified before. Altered expression of the miRNA regulators and their target genes in the self-renewal pathway was validated using quantitative PCR measurements.

### 4.1 Dysregulated miRNA pattern in chordoma

A large set (N = 126) of significantly dysregulated miRNAs in chordoma was identified in our study. A positive correlation between the expression changes for many miRNAs and their host genes suggests that several miRNAs are regulated predominantly at the transcriptional level, whereas for other miRNAs the transcriptional changes are counteracted or buffered by regulating the processing steps. Reciprocally regulated sets of differentially expressed genes and miRNAs, combined with miRNA target prediction highlighted a set of genes in chordoma that may be regulated by the altered miRNA network. Upregulated miRNAs appear to target elements of growth factor-mediated signaling networks, including the axon guidance pathway, which is often activated in other cancer types. Downregulated miRNAs may target a very different network: most of the enriched pathways are associated with the regulation of immune function, notably with immune suppression. For example, suppressed miR-665 expression may contribute to the activation of TNF signaling pathways, which may help the immune evasion of the chordoma cells through stabilizing the undifferentiated phenotype, and/or through immunoediting in the tumor microenvironment. Other putative miRNA-regulated genes in these pathways may reflect in part the immunosuppressive phenotype of chordoma cells, but also the presence of inactivated tumor-infiltrating immune cells. We hypothesize that the dominant miRNA regulators of chordoma are those with the most target genes in the differentially expressed gene set: the upregulated miR-182-5p, the miR-15/16/195/424/497-5p family, miR-148a, miR-140, miR-142, and miR-199a/b-5p, and the downregulated miR-3151-3p, miR-377-5p, and miR-665. miR-182-5p is a well-characterized oncomir promoting the aggressive phenotypes [48, 49, 50], and miR-377-5p acts as a tumor suppressor in different tumor types [51, 52]. The other dysregulated miRNAs, however, may have controversial, context-dependent effects in tumor cells. For instance, miR-424 may act either as a tumor suppressor or as an oncogene [53, 54], overexpression of miR-497 may contribute to chemoresistance or metastasis inhibition in different model systems [55, 56], and miR-223 may regulate chemoresistance differently in different tumor types [57, 58]. Similar to melanoma cells, we observed the reciprocal regulation of miR-126 (overexpressed) and miR-221/222 (downregulated) in chordomas [59].

### 4.2 Dysregulated miRNA/mRNA network can be associated with chordoma recurrence

A gene set targeted by several dysregulated miRNAs having a possible role in the self-renewal process of chordoma stem cells was identified through a detailed bioinformatic analysis. According to the cancer stem cell theory standard chemotherapy can kill most cells in a tumor; however, cancer stem cells are resistant and remain viable. Despite the small number of such cells in the tumor mass, they might be the cause of tumor recurrence, sometimes many years after the “successful” treatment of the primary tumor, which is very frequent in chordoma [60, 61]. A dysregulated network identified in our study includes 7 miRNAs that may regulate 5 target genes, all of which have potential roles in stem cell renewal. Significantly lower expression of LCOR (ligand-dependent nuclear receptor corepressor) gene in chordoma can be caused in part by the upregulation of seven miRNAs (miR-101, miR-142, miR-144, miR-148, miR-182, miR-199a, and miR-424), which are predicted or validated regulators of the LCOR mRNA. LCOR had been identified as a tumor suppressor in prostate cancer [62] and a recent study showed that the miR-199a-LCOR-IFN axis is a critical regulator maintaining the stem cell phenotype in breast cancer [63]. Some of the same upregulated miRNAs may target the elements of the Hippo signaling pathway - importantly, most of these miRNA-mRNA interactions have been validated in different model systems, as summarized in Supplementary Table 6. Five regulators of this pathway (TAOK1, TAOK2, MOB1A/B, and LATS1) were significantly suppressed in chordoma samples and two of them are predicted targets for upregulated miRNAs (TAOK1 for miR-142, and MOB1B for miR-148a and miR-182). All five genes are responsible for the inhibition of the YAP/TAZ complex, a central effector regulating tissue proliferation and self-renewal in normal and cancer tissues [64, 65, 66] (Figure 4). Although we did not observe altered expression of previously identified YAP/TAZ target genes in chordoma, it is well known that the target gene set of YAP/TAZ is highly context-dependent [66]. Other regulators of stemness, such as MYBL1 [67] and RECK [68] are also predicted targets for multiple upregulated miRNAs in our study, and, together with LCOR and YAP/TAZ may form a regulatory network maintaining the stem-cell phenotype in chordoma. Importantly, a recent study established that the YAP/TAZ pathway is indeed activated in chordoma tumors and contributes to cancer growth and stemness [69]. This study identified the YAP gene as one of the Brachyury targets, activated at the transcriptional level. Based on our results, the overexpressed oncomirs can also contribute to the activation of the pathway.

This feature of stemness regulation in chordoma tumors suggested by the bioinformatics analysis of the NGS results can be related to the unique origin of this tumor (ie. originated from notochordal remnants) and could explain the high rate of local recurrence as well as its resistance to approved chemotherapeutic agents.

### 4.3 Strength and limitations

The strength of our study is the integrated analysis of the noncoding and coding RNA expression profile, leading to the prediction of dominant miRNA regulators in chordoma, and the prediction of a novel miRNA-mRNA regulatory network. Another strength of our study is the validation of the miRNA-mRNA gene expression changes by RT-qPCR. Differences in miRNA expression profile may be variable depending on the location of chordoma and even among different chordoma cell lines [26]. The CH and NP samples in our study came from patients forming homogenous groups based on their clinical characteristics, to minimize the inter-donor heterogeneity. To avoid additional differences in the tumor transcriptomes, all chordoma samples were harvested from sacral chordoma tumors, and the patients did not receive any previous neo-adjuvant treatment. The small number of chordoma samples is a limitation of the study, but given that chordoma is a rare tumor (less than 1/million person/year new cases in Hungary), larger sample sets can be collected only in international collaborations, or in large countries. That said, even our small sample set corroborated previous findings, such as downregulation of miR-31 or overexpression of cytokeratin genes, and provides a reliable molecular portrait of chordoma.

Another important factor in the comparative chordoma studies is the type of the control sample. As chordoma originates from the notochord, the use of muscle [24, 28], fibroblast [29] or nasal turbinate tissue [70] as controls can lead to biased results, revealing the tissue-specific-differences of miRNA expression profiles, rather than tumor-specific differences. Similar to the majority of other authors [23, 26, 27, 30], we compared the transcriptome profile of chordoma to nucleus pulposus cells, which were derived from the single mature, non-dysplastic tissue type originating from notochord in humans. We obtained NP tissue samples for moderately degenerated intervertebral discs during fusion procedures to avoid the contamination of the NP tissue with annulus fibrous or cartilage parts, which is usually present in herniated disc materials. Several extraction methods were tested, but none yielded high-quality RNA suitable for NGS from surgical NP tissue samples – therefore, we used primary NP cell cultures as controls. The same setup was applied in the studies of Bayrak et al. [23] and Gulluoglu et al. [26] and our findings strongly supported their results: namely the overexpression of miR-140 and miR-148a, as well as the decreased expression of miR-31 and miR-222 in chordoma. In fact, miR-31 was the most downregulated miRNA in chordoma in our study.

Although our results are in good agreement with previous studies, which used similar sample sets but different RNA profiling methods [23, 26], further functional validation is needed to confirm our findings. This might be especially important in understanding the complex miRNA-dependent regulation of LCOR if novel therapeutical approaches are considered to target the LCOR-mediated IFN-responsiveness in chordoma. Also, different therapeutical approaches may be used to target the YAP/TAZ complex, affecting specific protein-protein interactions or downstream targets [71]. However, given the complexity and variability of the YAP/TAZ network, the best and critical targets may well be different in different tumor types. Importantly, our study revealed several regulatory miRNAs, the antimiR-based therapeutical suppression of which may lead to the reactivation of the Hippo pathway and the inhibition of YAP/TAZ and may target other stemness genes as well. Lastly, the clinical consequences of the ncRNA-regulated gene expression changes and the possible prognostic value of ncRNAs need further investigations on large, multicenter sample sets.

## 5 Conclusions

In chordoma development, crucial cancer driver genes have not been identified so far. Despite the identification of numerous genes with dysregulated expression in vitro or in vivo in chordoma (e.g. brachyury, EGFR, Ezrin, MMP-9, c-MET, FHIT, etc. [72]) conventional or targeted chemotherapeutic agents showed a partial response at best-case scenarios in clinical case-series. Significant genomic rearrangements were found in several chordoma samples in previous studies [17, 73] suggesting that the development and recurrence of the tumor can be related to the dysregulation of hundreds of genes and their complex regulatory networks such as non-coding RNAs. The recent development of high-throughput molecular profiling techniques (i.e. next-generation RNA sequencing) has further increased the possibility of performing comparative and integrated RNA profiling studies facilitating the detection of the alterations in the transcriptome and related regulatory networks [37, 74, 75]. Applying these techniques on surgical sacral chordoma tumor samples, several miRNAs with significant regulatory potential and a previously not identified miRNA/mRNA network involved in stemness regulation have been identified in our study. Molecular elements of these pathways are possible targets for future therapy development in this malignant, orphan disease.

## Declarations

### Author contribution statement

Arpad Bozsodi: Performed the experiments; Analyzed and interpreted the data; Contributed reagents, materials, analysis tools or data; Wrote the paper.

Beata Scholtz: Analyzed and interpreted the data; Contributed reagents, materials, analysis tools or data; Wrote the paper.

Gergo Papp: Performed the experiments; Analyzed and interpreted the data.

Zoltan Sapi: Contributed reagents, materials, analysis tools or data.

Adam Biczo: Analyzed and interpreted the data.

Peter Pal Varga; Aron Lazary: Conceived and designed the experiments; Wrote the paper.

### Funding statement

This work was supported by 10.13039/501100003549OTKA PD grant (104604) and the Eurospine Task Force Research (Grant no: ETFR 2012/001).

### Declaration of interests statement

The authors declare no conflict of interest.

### Data availability statement

Data associated with this study has been deposited at NCBI Gene Expression Omnibus under the accession number GSE209695.

### Additional information

No additional information is available for this paper.

## Appendix A Supplementary data

_Suppl_Fig_1A _Suppl_Fig_1A

_Suppl_Fig_1B _Suppl_Fig_1B

_Supplementary Figure 1_caption _Supplementary Figure 1_caption
