# Supplementary material for: Potential molecular mechanism in self-renewal is associated with miRNA dysregulation in sacral chordoma – A next-generation RNA sequencing study
Source: Heliyon. 2022 Aug 13;8(8):e10227. doi: 10.1016/j.heliyon.2022.e10227 (PMC9404356; doi:10.1016/j.heliyon.2022.e10227)
Supplement: _Supplementary Figure 1_caption [file mmc3.docx]

**Supplementary Figure 1 Predicted target genes of top up- (A) and downregulated (B) miRNAs in chordoma** Gray cells indicate Targetscan predictions of the miRNA-mRNA interaction. Anti-tumor or oncogenic classification was based on literature searches. Functional classification was based on PantherDB analysis and literature searches. Only the most prominent categories are shown – annotated genes outside these categories are lumped together in the "Mixed" category. Genes in the "mixed" category but without a connection with cancer are not shown. miRNAs with the highest number of predicted targets are highlighted in orange/pale orange. Targets of miRNAs in the same miRNA family are marked in the same column. Regulated miRNAs with only a few predicted targets are not shown.
