# Supplementary material for: Potential molecular mechanism in self-renewal is associated with miRNA dysregulation in sacral chordoma – A next-generation RNA sequencing study
Source: Heliyon. 2022 Aug 13;8(8):e10227. doi: 10.1016/j.heliyon.2022.e10227 (PMC9404356; doi:10.1016/j.heliyon.2022.e10227)
Supplement: _Supplementary Data [file mmc4.docx]

**RT-qPCR quantification of miRNAs and mRNAs**

1. The following miRNA were quantified with TaqMan™ Advanced miRNA assay, and the reverse transcription was done with the TaqMan™ Advanced miRNA RT kit (Applied Biosystem®), all according to the manufacturer’s instruction.

| **miRNA** | **TaqMan assay ID** |
| --- | --- |
| hsa-mir-148a-3p | 477814-mir |

1. The following miRNAs and mRNAs were quantified with standard TaqMan™ miRNA and Gene Expression assays, and the reverse transcription (RT) was done using the High Capacity cDNA Reverse Transcription Kit (Applied Biosystem®). miRNA specific RT primers were mixed in equal molar ratios, and 9 μL of the primer mix was used in the RT reaction, in a final volume of 15 μL. RT reactions for detecting mRNAs were done with random hexamer priming. The cDNA samples were diluted 5-fold, and 2 μL of the diluted samples were used for qPCR, using TaqMan™ Universal qPCR mastermix, and 10 μL final reaction volume. PCR cycle parameters were as follows: 10 min at 95 °C, then 40 cycles of 15 sec at 95 °C and 60 sec at 60 °C. Each gene was measured in duplicates.

| **miRNA** | **TaqMan assay ID** |
| --- | --- |
| miR-182-5p | 002334_mir |
| miR-142-3p | 000464_mir |
| miR-144-3p | 002676_mir |

| **mRNA** | **TaqMan assay ID** |
| --- | --- |
| TAOK1 | Hs01020477_m1 |
| TAOK2 | Hs00191170_m1 |
| MOB1B | Hs01398837_m1 |
| LATS1 | Hs01125524_m1 |
| MOB1A | Hs00964416_m1 |
| LCOR | Hs00287120_m1 |
