# Supplementary material for: Potential molecular mechanism in self-renewal is associated with miRNA dysregulation in sacral chordoma – A next-generation RNA sequencing study
Source: Heliyon. 2022 Aug 13;8(8):e10227. doi: 10.1016/j.heliyon.2022.e10227 (PMC9404356; doi:10.1016/j.heliyon.2022.e10227)
Supplement: _Supplementary_Table 2 [file mmc6.docx]

**Supplementary Table 2**

**Quantification and quality control of RNA samples used in the study.** RNA concentration was determined by NanoDrop analysis.

| **Sample ID** | **Sample origin** | **RNA ng/ul** | **260/280** | **260/230** | **Agilent RIN** |
| --- | --- | --- | --- | --- | --- |
| CH4 | Chordoma surgical specimen | 223.19 | 2.08 | 2.32 | 8.2 |
| CH6 |  | 468.01 | 1.95 | 1.95 | 8.2 |
| CH7 |  | 462.8 | 1.94 | 1.27 | 8.3 |
| CH8 |  | 3262.68 | 1.93 | 1.96 | 8.8 |
| CH10 |  | 578.77 | 2.03 | 1.43 | 8.6 |
| CH11 |  | 1357.92 | 2 | 1.82 | 7.3 |
| CH12 |  | 938.61 | 2.03 | 1.78 | 8.0 |
| CH13 |  | 1045.25 | 2.06 | 1.54 | 7.9 |
| NP5 | Nucleus pulposus cell culture | 233.58 | 2.07 | 2 | 8.1 |
| NP6 |  | 218.29 | 2.07 | 1.99 | 9.2 |
| NP7 |  | 311.51 | 2.06 | 1.91 | 9.1 |
| NP9 |  | 900.41 | 2.15 | 2.12 | 10 |
| NP10 |  | 832.77 | 2.15 | 2.11 | 10 |
| NP11 |  | 599.07 | 2.13 | 1.36 | 10 |
| NP12 |  | 209.48 | 2.09 | 2.07 | 7.9 |
| NP13 |  | 687.45 | 2.13 | 2.06 | 9.6 |
