# Supplementary material for: Potential molecular mechanism in self-renewal is associated with miRNA dysregulation in sacral chordoma – A next-generation RNA sequencing study
Source: Heliyon. 2022 Aug 13;8(8):e10227. doi: 10.1016/j.heliyon.2022.e10227 (PMC9404356; doi:10.1016/j.heliyon.2022.e10227)
Supplement: _Supplementary_Table_4 [file mmc8.docx]

Supplementary Table 5.

Functional classification analysis of the predicted targets of upregulated and downregulated miRNAs in chordoma, based on Panther DB analysis.
