# Supplementary material for: Potential molecular mechanism in self-renewal is associated with miRNA dysregulation in sacral chordoma – A next-generation RNA sequencing study
Source: Heliyon. 2022 Aug 13;8(8):e10227. doi: 10.1016/j.heliyon.2022.e10227 (PMC9404356; doi:10.1016/j.heliyon.2022.e10227)
Supplement: _Supplementary_Table 5 [file mmc9.docx]

**Supplementary Table 3**

**Most important candidate target genes of differentially expressed miRNAs in chordoma**

| **Predicted target gene** | **log2FoldChange of target gene** | **miRNA** | **Cumulative weighted context++ score** |
| --- | --- | --- | --- |
| LCOR | -3.77 | hsa-miR-424-5p | -0.32 |
|  |  | hsa-miR-101-3p | -0.78 |
|  |  | hsa-miR-142-3p | -0.66 |
|  |  | hsa-miR-144-3p | -0.73 |
|  |  | hsa-miR-199a-5p | -0.67 |
| MOB1B | -3.73 | hsa-miR-148a-3p | -0.39 |
|  |  | hsa-miR-182-5p | -0.34 |
| TAOK1 | -4.50 | hsa-miR-142-3p | -0.67 |
| MITF | -3.94 | hsa-miR-101-3p | -0.35 |
|  |  | hsa-miR-144-3p | -0.3 |
|  |  | hsa-miR-148a-3p | -0.74 |
|  |  | hsa-miR-182-5p | -0.73 |
| MYBL1 | -3.61 | hsa-miR-424-5p | -0.5 |
|  |  | hsa-miR-144-3p | -0.49 |
| RECK | -2.69 | hsa-miR-497-5p | -0.45 |
|  |  | hsa-miR-182-5p | -0.46 |
| ABL2 | -3.44 | hsa-miR-15a-5p | -0.37 |
|  |  | hsa-miR-143-3p | -0.39 |
|  |  | hsa-miR-148a-3p | -0.42 |
| CAMK2N1 | -3.48 | hsa-miR-140-5p | -0.36 |
|  |  | hsa-miR-182-5p | -0.57 |
|  |  | hsa-miR-450b-5p | -0.78 |
| DST | -5.26 | hsa-miR-335-3p | -0.9 |
|  |  | hsa-miR-126-5p | -0.33 |
| ECE1 | -3.36 | hsa-miR-140-3p | -0.52 |
|  |  | hsa-miR-199a-5p | -0.63 |
| FAM73A | -3.36 | hsa-miR-424-5p | -0.55 |
|  |  | hsa-miR-101-3p | -0.46 |
|  |  | hsa-miR-142-3p | -0.4 |
|  |  | hsa-miR-182-5p | -0.44 |
| HMGA2 | -9.19 | hsa-miR-16-5p | -0.55 |
|  |  | hsa-miR-142-3p | -0.72 |
|  |  | hsa-miR-204-5p | -0.44 |
| JMJD1C | -4.87 | hsa-miR-96-5p | -0.58 |
|  |  | hsa-miR-148a-5p | -0.32 |
|  |  | hsa-miR-182-5p | -0.77 |
| MIPOL1 | -2.74 | hsa-miR-497-5p | -0.43 |
|  |  | hsa-miR-140-5p | -0.34 |
|  |  | hsa-miR-192-5p | -0.46 |
| NBEAL1 | -4.58 | hsa-miR-126-5p | -0.62 |
|  |  | hsa-miR-335-3p | -0.59 |
| NDP | -6.90 | hsa-miR-424-5p | -0.47 |
|  |  | hsa-miR-148a-3p | -0.4 |
|  |  | hsa-miR-223-3p | -0.43 |
| PDPN | -4.14 | hsa-miR-182-5p | -0.37 |
|  |  | hsa-miR-199a-5p | -0.7 |
| RASA1 | -2.07 | hsa-miR-182-5p | -0.63 |
|  |  | hsa-miR-223-3p | -0.39 |
| RGS17 | -3.17 | hsa-miR-144-3p | -0.54 |
|  |  | hsa-miR-182-5p | -1 |
| SPIN1 | -2.29 | hsa-miR-96-5p | -0.86 |
|  |  | hsa-miR-182-5p | -0.44 |
| SPTSSB | -4.40 | hsa-miR-96-5p | -0.43 |
|  |  | hsa-miR-182-5p | -0.45 |
|  |  | hsa-miR-450b-5p | -0.57 |
| TFRC | -3.04 | hsa-miR-15a-5p | -0.46 |
|  |  | hsa-miR-148a-5p | -0.41 |
|  |  | hsa-miR-335-3p | -0.37 |
|  |  | hsa-miR-450b-5p | -0.75 |
| RICTOR | -3.62 | hsa-miR-142-3p | -0.72 |
| ARHGAP21 | -4.99 | hsa-miR-199b-5p | -0.66 |
| ARHGEF12 | -4.61 | hsa-miR-182-5p | -0.74 |
| HMCN1 | -4.96 | hsa-miR-199a-5p | -0.69 |
| HOXB6 | -3.83 | hsa-miR-126-5p | -0.64 |
| NEK10 | -4.62 | hsa-miR-497-5p | -0.75 |
| PRKACB | -3.42 | hsa-miR-182-5p | -0.76 |
| PTPN3 | -3.97 | hsa-miR-424-5p | -0.64 |
| NFATC2 | 4.41 | hsa-miR-431-3p | -0.35 |
|  |  | hsa-miR-665 | -0.24 |
| HIF3A | 6.73 | hsa-miR-377-5p | -0.63 |
|  |  | hsa-miR-485-5p | -0.64 |
| PRELP | 2.33 | hsa-miR-31-5p | -0.4 |
|  |  | hsa-miR-665 | -0.67 |
| AKR1B10 | 7.73 | hsa-miR-3151-3p | -0.89 |
| C21orf119 | 2.19 | hsa-miR-539-3p | -0.63 |
| HEYL | 7.35 | hsa-miR-665 | -0.62 |
| IL15 | 2.55 | hsa-miR-3151-3p | -0.82 |
| LYPD6 | 4.84 | hsa-miR-431-5p | -0.76 |
| MYEOV2 | 2.53 | hsa-miR-431-3p | -1.15 |
| MYO5C | 3.64 | hsa-miR-377-5p | -0.9 |
| P2RY13 | 7.60 | hsa-miR-431-3p | -0.7 |
| RADIL | 4.65 | hsa-miR-665 | -0.72 |
| SH2D1A | 5.83 | hsa-miR-31-5p | -0.87 |
| SYNGR2 | 2.92 | hsa-miR-665 | -0.61 |
| YPEL2 | 2.41 | hsa-miR-3151-3p | -0.68 |
