# Supplementary material for: Potential molecular mechanism in self-renewal is associated with miRNA dysregulation in sacral chordoma – A next-generation RNA sequencing study
Source: Heliyon. 2022 Aug 13;8(8):e10227. doi: 10.1016/j.heliyon.2022.e10227 (PMC9404356; doi:10.1016/j.heliyon.2022.e10227)
Supplement: _Suppl_Fig_1A [file mmc1.pdf]

Category	Gene	Type (Anti-tumor / Oncogenic)	miR-96-5p	miR-101-3p.1	miR-126-3p/5p	miR-140-3p/5p	miR-142-3p/5p	miR-143-3p	miR-144-3p/5p	miR-148a-3p/5p/148b-3p	miR-15-5p/16-5p/195-5p/424-5p/497-5p	miR-182-5p	miR-199a/b-5p	miR-204-5p	miR-335-3p
Transcription factors	BNC2	Anti-tumor					■								
	E2F7	Anti-tumor									■				
	APBB1	Anti-tumor							■						
	EBF3	Anti-tumor										■			
	FOXP2	Anti-tumor	■												
	HOXB6	Anti-tumor			■										
	MITF	Anti-tumor		■					■	■		■			
	RB1	Anti-tumor													
	REST	Anti-tumor				■									
	ZNF24	Anti-tumor		■											
	PPARGC1A	Anti-tumor											■		
	LCOR	Anti-tumor		■			■		■		■		■		
	ETV5	Oncogenic		■											
	FOSL1	Oncogenic									■				
	MYBL1	Oncogenic							■		■				
	SIX4	Oncogenic								■					
	PAXBP1												■		
	PLAG1										■				
	ZNF286A									■					
Signal transduction	LIF	Anti-tumor				■									
	AKAP2	Anti-tumor												■	
	PRKACB	Anti-tumor										■			
	PRKAR1A	Anti-tumor										■			
	PRKAR2A	Anti-tumor									■				
	KANK2	Anti-tumor			■										
	NDP	Anti-tumor								■	■				
	NPTX1	Anti-tumor								■		■			
	CAMK2N1	Anti-tumor				■						■			
	RPRD1A	Anti-tumor					■								
	SOCS4	Anti-tumor													
	PTPN14	Anti-tumor								■					
	PTPN3	Anti-tumor									■				
	KANK1	Anti-tumor									■				
	PIK3C2A	Oncogenic								■					
	DCBLD2	Oncogenic		■											
	SEMA6D										■				
	MOB1B									■		■			
	PPP4R4														
	ADAM9				■										
	CNTNAP1										■				
	NCS1										■				
G proteins or regulator of	ARHGAP21	Anti-tumor											■		
	ARHGEF12	Anti-tumor										■			
	RASA1	Anti-tumor										■			
	RASA2	Anti-tumor										■			
	RASSF8	Anti-tumor									■				
	RGS17	Anti-tumor							■			■			
	AKAP13	Oncogenic				■									
	RGS3	Oncogenic			■										
	SOS1	Oncogenic								■					
	ARL10										■				
	GNG12					■									
	RALGAPA1												■		
	SBF2									■					
	TBC1D19								■						
Chromatin associated	ATXN1L	Anti-tumor		■							■				
	ANKRD11	Anti-tumor		■			■								
	ARID2	Anti-tumor							■						
	JMJD1C	Anti-tumor	■							■		■			
	SPIN1	Oncogenic	■									■			
	HMGA1	Oncogenic					■				■				
	HMGA2	Oncogenic					■				■			■	
	ARID5B						■								
	ASH1L						■				■				
	CHD2										■				
	KMT2E								■						
	SRCAP					■									
Protein kinases	SMG1	Anti-tumor													■
	NEK1	Anti-tumor													■
	NEK10	Anti-tumor									■				
	ABL2	Oncogenic						■		■	■				
	CDKL1	Oncogenic							■						
	FER	Oncogenic											■		
	SLK	Oncogenic													■
	YES1	Oncogenic										■			
	GSK3B	Oncogenic											■		
	IRAK2	Oncogenic									■				
	MAPK1	Oncogenic						■							
	MYLK	Oncogenic					■				■				
	PDK1	Oncogenic	■												
	DSTYK									■					
	PSD3							■							
	TAOK1						■								
Mixed categories	BAG5	Anti-tumor									■				
	VWA8	Anti-tumor												■	
	PCDH10	Anti-tumor				■									
	CLDN11	Anti-tumor		■											
	NIPBL	Anti-tumor													
	PDS5B	Anti-tumor													■
	RECK	Anti-tumor									■	■			
	MIPOL1	Anti-tumor				■					■				
	KIF1B	Anti-tumor				■					■				
	SUN1	Anti-tumor											■		
	DDX3Y	Anti-tumor									■				
	PDPN	Oncogenic										■	■		
	APLP2	Oncogenic													■
	RICTOR	Oncogenic					■								
	CPEB4	Oncogenic								■					
	CLIC4	Oncogenic					■								
	NAALADL2	Oncogenic											■		
	PHF20L1	Oncogenic	■												
	TACC1	Oncogenic									■				
	PFN2	Oncogenic				■									
	ACSL4	Oncogenic												■	
	ADAM17	Oncogenic								■					
	ASPH	Oncogenic								■					
	ECE1	Oncogenic				■							■		
	PC	Oncogenic						■				■			
	IL17RD	Oncogenic				■									
	ITPR1	Oncogenic										■			
	MET	Oncogenic										■			
	ROBO1	Oncogenic								■					
	BDNF	Oncogenic									■				
	EREG	Oncogenic			■										
